# A risk scoring system based on tumor microenvironment cells to predict prognosis and immune activity in triple-negative breast cancer

**DOI:** 10.1007/s12282-021-01326-w

**Published:** 2022-01-21

**Authors:** Anli Yang, Minqing Wu, Mengqian Ni, Lijuan Zhang, Mingyue Li, Peijun Wei, Yonggang Yang, Weikai Xiao, Xin An

**Affiliations:** 1 State Key Laboratory of Oncology in South China, Collaborative Innovation Center for Cancer Medicine, Guangzhou, 510060 Guangdong China; 2 Department of Breast Oncology, Sun Yat-sen University Cancer Center, Guangzhou, China; 3 Department of Cancer Prevention, Sun Yat-sen University Cancer Center, Guangzhou, China; 4 Department of Medical Oncology, Sun Yat-sen University Cancer Center, Guangzhou, China; 5 Department of Pathology and Laboratory Medicine, Perelman School of Medicine, University of Pennsylvania, Philadelphia, PA USA; 6 Department of Intensive Care Unit, Sun Yat-sen University Cancer Center, Guangzhou, China; 7 Department of Anesthesiology, Sun Yat-sen University Cancer Center, Guangzhou, China; 8 Department of Breast Cancer, Cancer Center, Guangdong Provincial People’s Hospital, Guangdong Academy of Medical Sciences, Guangzhou, 510080 Guangdong China

**Keywords:** Triple-negative breast cancer, Prognostic scoring system, Immunotherapy

## Abstract

The tumor microenvironment (TME) interacting with the malignant cells plays a vital role in cancer development. Herein, we aim to establish and verify a scoring system based on the characteristics of TME cells for prognosis prediction and personalized treatment guidance in patients with triple-negative breast cancer (TNBC). 158 TNBC samples from The Cancer Genome Atlas (TCGA) were included as the training cohort, and Molecular Taxonomy of Breast Cancer International Consortium (METABRIC) (*N* = 297), as well as GSE58812 (*N* = 107), were included as the validation cohort. The enrichment scores of 64 immune and stromal cells were estimated by the xCell algorithm. In the training cohort, cells with prognostic significance were found out using univariate Cox regression analysis and further applied to the random survival forest (RSF) model. Based on the scores of M2 macrophages, CD8^+^ T cells, and CD4^+^ memory T cells, a risk scoring system was constructed, which divided TNBC patients into 4 phenotypes (M2^low^, M2^high^CD8^+^T^high^CD4^+^T^high^, M2^high^CD8^+^T^high^CD4^+^T^low^, and M2^high^CD8^+^T^low^). Furthermore, types 1 and 2 patients were merged into the low-risk group, while types 3 and 4 patients were in the high-risk group. The low-risk group had superior survival outcomes than the high-risk one, which was further confirmed in the validation cohort. Moreover, in the low-risk group, immune-related pathways were significantly enriched, and a higher level of antitumoral immune cells and immune checkpoint molecules, including PD-L1, PD-1, and CTLA-4, could be observed. Additionally, consistent results were achieved in the SYSUCC cohort when the scoring system was applied. In summary, this novel scoring system might predict the survival and immune activity of patients and might serve as a potential index for immunotherapy.

**Supplementary Information:**

The online version contains supplementary material available at 10.1007/s12282-021-01326-w.

## Introduction

Female breast cancer has become the most commonly diagnosed cancer worldwide in 2020 [33]. Even though triple-negative breast cancer (TNBC) only accounts for 15–20% of all breast cancers, the aggressive behavior and lacking effective target agents make it a great challenge in clinical practice. Compared with other subtypes of breast cancer, TNBC shows a higher rate of metastasis and inferior prognosis [9, 19].

As the most important advance in the treatment of TNBC in recent years, immunotherapy using immune checkpoint inhibitors (ICIs) shows promising efficacy both in early-stage and metastatic TNBC [6, 31]. However, inconsistent results have been observed by various studies. The Impassion130 trial showed the addition of PD-L1 inhibitor atezolizumab to first-line nab-paclitaxel significantly improved progression-free survival (PFS) and overall survival (OS) in PD-L1-positive patients [30]. The KEYNOTE-355 trial reported that in PD-L1-positive TNBC, the PD-1 inhibitor pembrolizumab combined with first-line chemotherapy could significantly increase the benefit of PFS [11]. However, the most recent Impassion131 trial failed to exhibit the benefit of atezolizumab combined with paclitaxel even in PD-L1-positive patients [14]. Besides, the disparity in the benefits of ICIs neoadjuvant administration was also demonstrated in the early-stage TNBC. Both KEYNOTE-522 trial and Impassion031 trial showed the combination ICIs and chemotherapy increased pathological complete response (pCR) rates, while the NeoTRIPaPDL1 phase III trial failed to show any pCR improvement when atezolizumab was added to neoadjuvant chemotherapy [1, 14].

Factors such as steroid premedication and different chemotherapy backbones may contribute to these conflicting results, but one of the most important factors is the poor predictive value of PD-L1 [14, 34]. There are discrepancies among various PD-L1 detection assays, as well as differential expressions between primary and metastatic sites [28, 29]. A proportion of PD-L1-positive patients do not respond to ICIs while some PD-L1-negative patients do [20]. The above-mentioned issues limit PD-L1 as a biomarker for immunotherapy efficacy prediction. Herein, searching for a more reliable predictive marker is urgently needed.

Previous studies mainly focused on molecular biomarkers of malignant cells, such as mRNA panels and protein signatures, to find more specific TNBC classification methods and construct prognostic prediction models. However, more and more pieces of evidence show that the tumor microenvironment (TME) plays a crucial role in tumor initiation, invasion, and metastasis, including various immune cells, stromal cells, and many other cells that interact with the malignant ones. Recently, researchers pointed out the importance of TME in response to immunotherapy [15]. Therefore, analyzing the cellular heterogeneity of TME may provide more detailed information for precise classification of TNBC and predicting the applicability of immunotherapy.

In this study, 64 immune and stromal cells in the TME of TNBC were screened for prognostic relevance and applied to model construction in silico. We evaluated the distinguishing efficacy of our scoring system in identifying patients’ survival outcomes and immune checkpoint molecule expressions using bioinformatics analysis. Additionally, we confirm our findings in tissue samples from the SYSUCC cohort by immunohistochemical (IHC) staining to facilitate its clinical application.

## Materials and methods

### Data acquisition

**Fig. 1:**
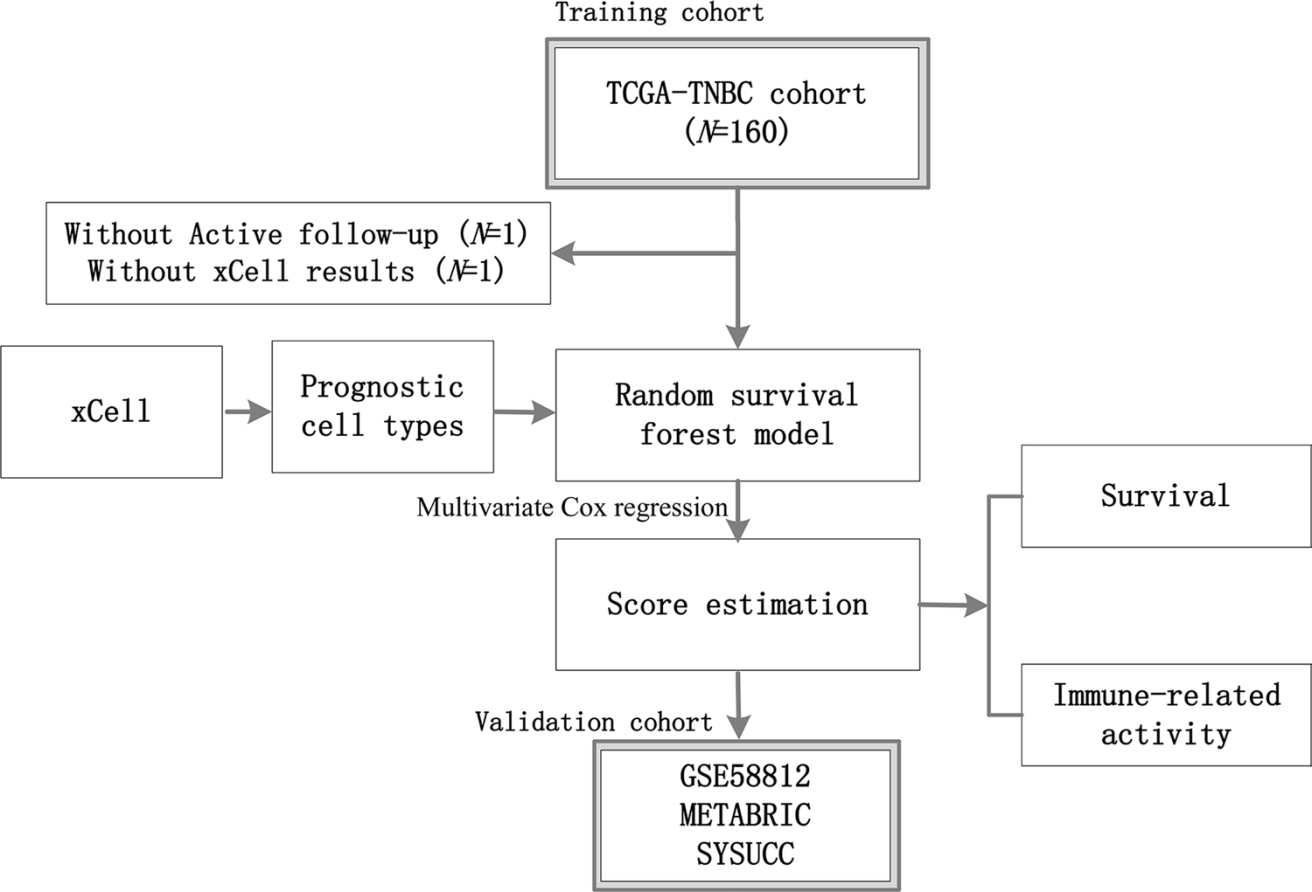
Study design. A risk score system was constructed in the training cohort (TCGA, *N* = 158) and further validated in the validation cohorts (GSE58812, METABRIC, SYSUCC)

Our study design is displayed in Fig. 1. The inclusion criteria for patients from The Cancer Genome Atlas (TCGA), Molecular Taxonomy of Breast Cancer International Consortium (METABRIC), and the Gene Expression Omnibus (GEO) datasets are as follows: (a) histologically diagnosed with triple-negative breast cancer (TNBC); (b) available for clinical data, such as overall survival (OS) data; and (c) available for xCell-derived score matrix. Patients without active follow-up were excluded. Through preliminary screening, a total of 158 patients from the TCGA dataset were included as the training cohort, and the validation cohort consisted of patients from the METABRIC database (*N* = 297) and GSE58812 (*N* = 107). The TCGA dataset was obtained from TCGA using gdc-client. METABRIC data were downloaded from cBioPortal under the guidelines of the website (http://www.cbioportal.org/) [12]. The mRNA expression matrix of GSE58812 was downloaded from the GEO dataset (https://www.ncbi.nlm.nih.gov/geo/) [21].

### Collection of specimens

For the Sun Yat-sen University Cancer Center (SYSUCC) cohort, the following criteria were used for the patient selection: (a) pathologically diagnosed with TNBC and underwent curative surgery; (b) the molecular subtypes were determined by immunohistochemistry (IHC) staining, and Her2 status was further validated using fluorescence in situ hybridization (FISH) if indecipherable in IHC; (c) the staging of the patient was determined according to the AJCC 7th TNM staging system; (d) without a second primary malignancy; (e) no neoadjuvant chemotherapy or immunotherapy before operation. Also, patients without active follow-up were excluded. As a result, a total of 51 patients diagnosed between 2009 and 2011 were enrolled in the SYSUCC cohort. This study was conducted under the guidance of the Helsinki Declaration and approved by the institutional review committee of SYSUCC.

### Immunohistochemical staining

IHC was performed on tissue specimens collected from the SYSUCC cohort. The specimens were fixed with formalin and then embedded with paraffin. The embedded specimens were cut into 3um sections before staining and further undergo the antigen-retrieval procedure at 98 °C in citrate buffer (pH 6.0) for 10 min. To reduce the possibility of the nonspecific staining caused by endogenous peroxidase, sections were placed in the 3% hydrogen peroxide and incubated for 10–15 min, followed by a rinse with buffer solution twice. The sections were further incubated with diluted primary antibody overnight at 4 °C. Rinse twice with TBS, then incubate with HRP Polymer for 30 min at room temperature. The 3,3′-diaminobenzidine (DAB) system was used as the chromogen and the sections were counterstained with hematoxylin[35]. The slides were observed using the NIKON ECLIPSE 80i microscope. IHC results were evaluated by two independent pathologists who were blinded to the clinical data.

### Prognosis-related cell selection and model construction

We used the xCell algorithm to accurately identify the enrichment of multiple cells in the TME of TNBC. xCell is a method for cell-type enrichment analysis using single-sample Genome Set Enrichment Analysis (ssGSEA), which uses a spill-over compensation technique to reduce dependencies between closely related cell types. And it integrates the advantages of gene set enrichment with deconvolution approaches and covers a variety of immune and stromal cells. Through calculating ssGSEA scores for gene signatures and averaging the scores of all signatures corresponding to cell types, information on the enrichment scores of 64 cell types were extracted from all samples[3]. We found 6 cells with prognostic significance by the univariate Cox regression analysis and enrolled them into the random survival forest (RSF) model. Before undergoing the univariate Cox survival analysis, patients were divided into two groups based on the median of xCell scores. The log-rank test was used to further analyze the correlation between prognostic cells and survival probability. The RSF model was constructed to select the key cells and determine their comprehensive roles in survival. According to the decision tree with distinct survival, 158 TNBC patients in the TCGA cohort were divided into 4 immunophenotypes based on the 3 main prognostic cells.

### Establishment of the scoring system

Multivariate Cox regression was applied to estimate the risk score using the generated coefficients and corresponding expression. Furthermore, the patients were divided into the low-risk and high-risk groups based on their risk scores. Time-dependent receiver operating characteristic (ROC) analysis was performed and the areas under curve (AUC) at different time points were calculated to assess the discrimination value of the score. Survival analyses, including log-rank test, univariate and multivariate Cox regression models were adopted. The fractions of tumor-infiltrating immune cells were assessed by uploading the expression matrices to the cell-type identification which estimated relative subsets of RNA transcripts and calculated the scores based on LM22 signatures with 1000 permutations.

### Statistical analysis

The univariate and multivariate Cox regression was performed to confirm the independent predictors for OS using the “survival” package in R software[36]. The threshold was determined using the “randomForestSRC” package in R software. The survival curves related to prognostic analysis were prepared by the Kaplan–Meier method, and log-rank tests were used to determine the significance of survival differences. A time-dependent ROC analysis and the AUC were adopted to evaluate the accuracy of survival prediction by using the “timeROC” package in R software[8]. The significance of differences in the fractions of immune cells was estimated by the Wilcoxon test. All reported *P* values have corresponded to bilateral tests and the *P* value < 0.05 indicated that the difference between the groups was statistically significant. All statistical analyses were performed on R software version 3.5.3 (https://www.r-project.org/).

## Results

### Selection of prognosis-related cells

**Fig. 2:**
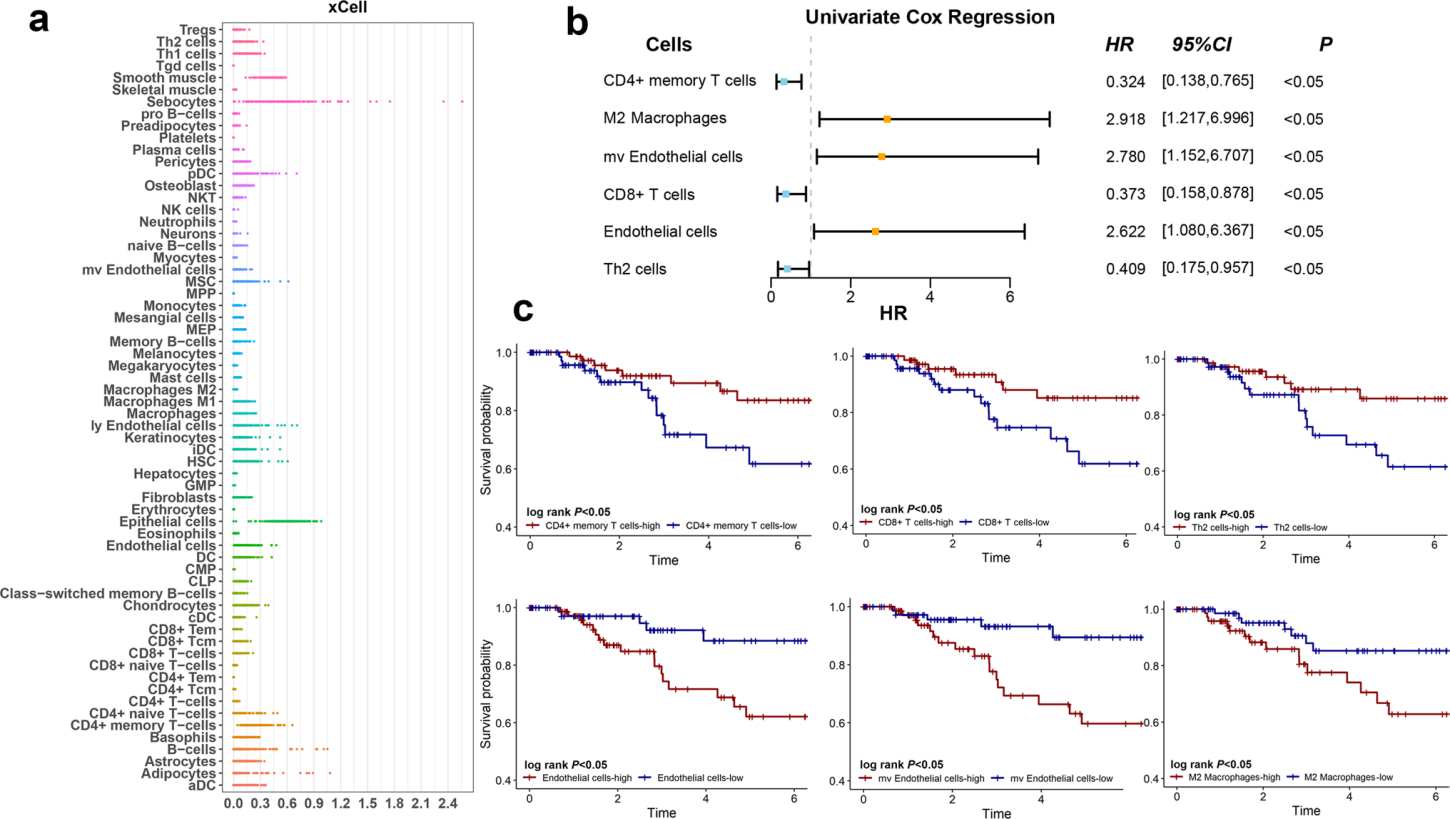
Selection of prognosis-related cells and survival analysis. **a** The profile displayed the scores of 64 immune and stromal cell types in the tumor microenvironment of TNBC from the TCGA cohort, which is extracted by the xCell algorithm. **b** 6 types of cells were selected by the univariate Cox regression analysis. **c** Kaplan–Meier analyses of 6 cell types in 562 samples from TNBC patients from TCGA, METABRIC, and GSE58812. A log-rank test was used for data analysis

The contents of 64 immune and stromal cells in the TME of TNBC were calculated through the xCell algorithm. An overview of the scores in the training cohort was shown in Fig. 2a. To further determine the prognostic value of these cells, a univariate Cox regression model was applied. CD4^+^ memory T cells *(HR* [hazard ratio] 0.324, *95%CI* [confidence interval] 0.138–0.765, *P* < 0.05), M2 macrophages (*HR* 2.918, *95%CI* 1.217–6.996, *P* < 0.05), mv endothelial cells (*HR* 2.780, *95%CI* 1.152–6.707, *P* < 0.05), CD8^+^T cells (*HR* 0.373, *95%CI* 0.158–0.878, *P* < 0.05), endothelial cells (*HR* 2.622, *95%CI* 1.080–6.376, *P* < 0.03), and Th2 cells (*HR* 0.409, *95%CI* 0.175–0.957, *P* < 0.05) were identified (Fig. 2b). Kaplan–Meier analysis of these 6 kinds of cells were shown in Fig. 2c. The log-rank test of OS showed consistent results that higher scores of CD4^+^ memory T cells, CD8^+^ T cells, and Th2 cells were associated with better prognosis, while M2 macrophages, endothelial cells, and mv endothelial cells did the opposite.

### Model construction and patient classification

**Fig. 3:**
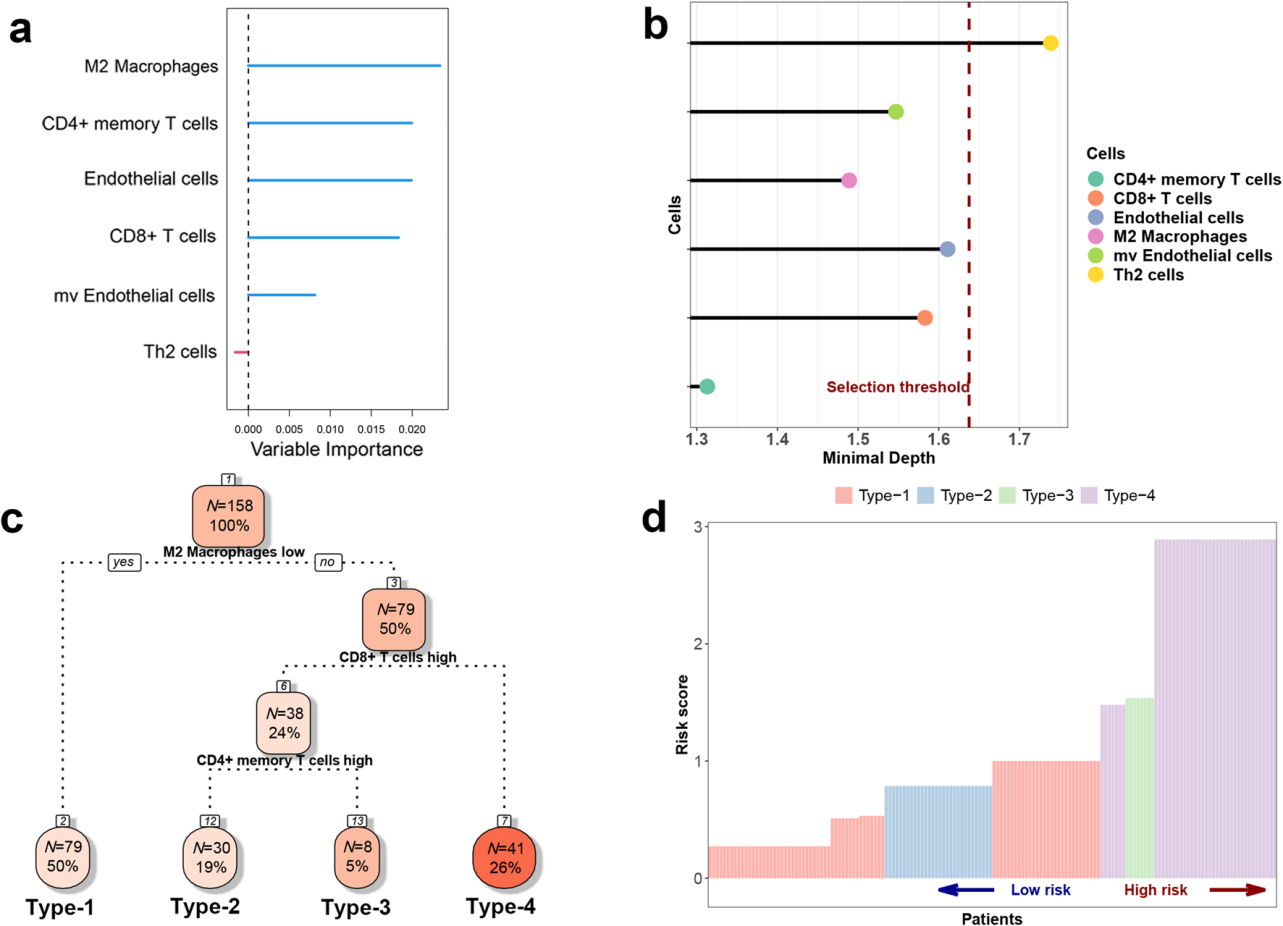
Model construction and patient classification. **a** The importance value analysis of the 6 candidate cells in the random survival forest model. **b** The minimal depth analysis of the 6 candidate cells. The vertical line in red means the selection threshold. **c** The decision tree exhibited that 158 TNBC patients were divided into 4 phenotypes by the scores of 3 cell types. **d** The risk score of each patient was shown. Columns represent samples sorted by the score from low to high

To comprehensively quantify the TME of TNBC and better predict patients’ prognosis, the random survival forest (RSF) model was applied to construct a novel scoring system. The above-mentioned 6 prognosis-related cells were further selected by variable importance (VIMP) and minimal depth analysis for the model construction (Fig. 3a, b). During the VIMP analysis, M2 macrophages showed the highest variable importance, followed by CD4^+^ memory T cells. However, Th2 cells should be discarded from the model for their negative VIMP. Through the minimum depth analysis, we found that the minimum depth of CD4^+^ memory T cells is the smallest, indicating that they are the most powerful in prognostic prediction. Th2 cells have the largest minimum depth and exceed the selection threshold, so they are excluded. As is well known, the endothelial cell subtypes are hard to distinguish by IHC assay. To facilitate clinical application, only CD4^+^ memory T cells, CD8^+^ T cells, and M2 macrophages were selected to build up the model. The decision tree was applied for phenotypes classification. Accordingly, patients were divided into 4 phenotypes based on the scores of these 3 kinds of cells. Patients with a M2^low^ feature were classified as type 1 (*N* = 79, 50%), M2^high^CD8^+^T^high^CD4^+^T^high^ as type 2 (*N* = 30, 19%), M2^high^CD8^+^T^high^CD4^+^T^low^ as type 3 (*N* = 8, 5%), and M2^high^CD8^+^T^low^ as type 4 (*N* = 41, 26%) (Fig. 3c). To further quantify, the score of each patient was evaluated by the multivariate Cox regression model. The scores of types 3 and 4 were significantly higher than those of types 1 and 2, which was consistent with the results obtained by the RSF model, indicating that types 3 and 4 patients are in a relatively high-risk state (Fig. 3d). Hence, types 1 and 2 patients were merged into the low-risk group, while types 3 and 4 patients were in the high-risk group.

### Prognostic and diagnostic value of the classifier

**Fig. 4:**
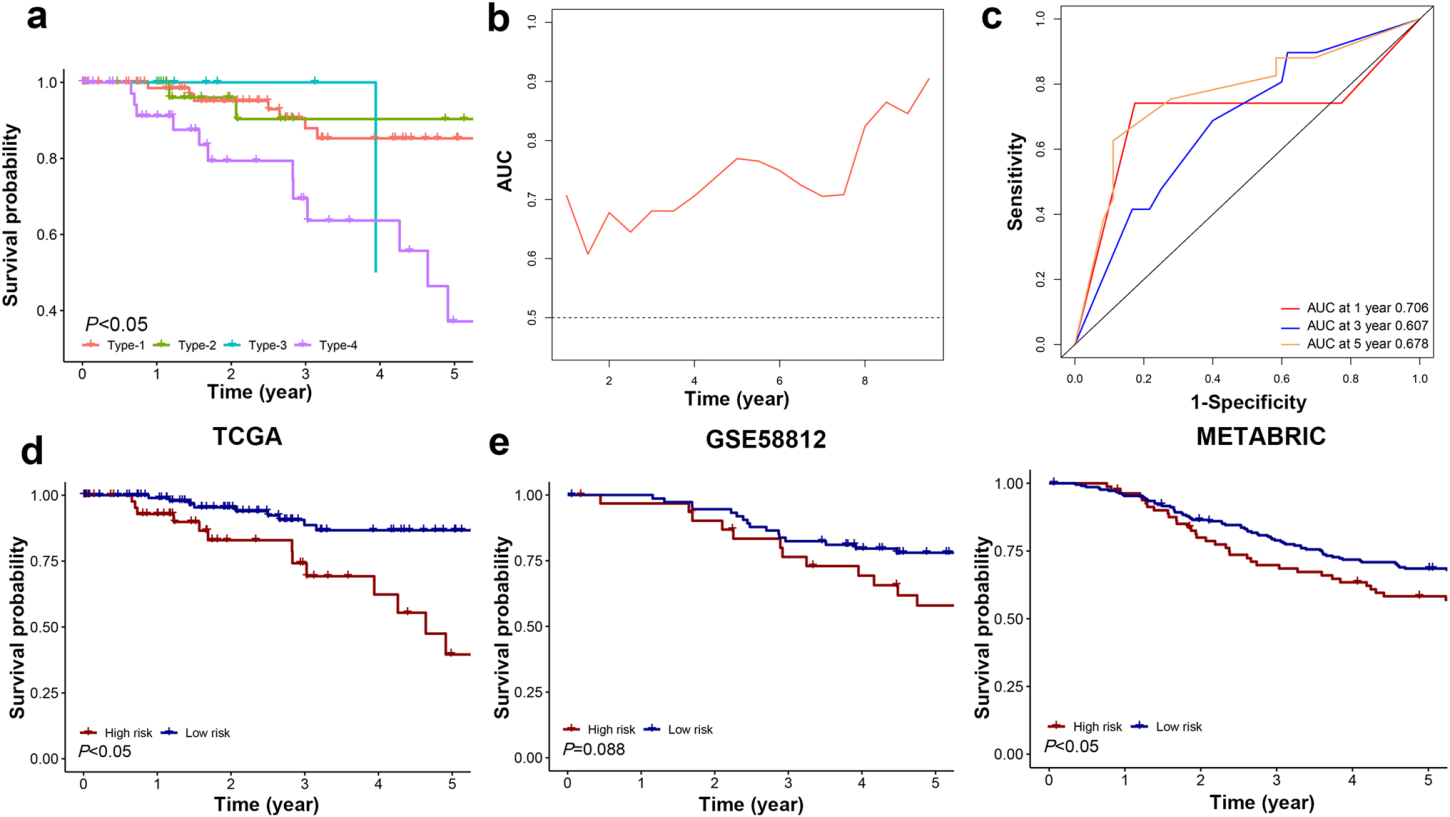
Prognostic and diagnostic value of the classifier. **a** Kaplan–Meier analysis of OS among the 4 phenotypes. **b** Time-dependent ROC analysis of the classifier regarding OS in patients with TNBC. **c** Receiver operating characteristic (ROC) curves showed the diagnostic value of 1, 3, and 5 years after diagnosis. **d** Kaplan–Meier analysis of OS between the 2 groups in the TCGA cohort. **e** Kaplan–Meier analysis of OS between the 2 groups in the validation cohorts. A log-rank test was used for data analysis

The analysis above was used to construct a comprehensive indicator and identify 4 phenotypes and 2 subgroups of TNBC patients with distinctive features. To verify the prognostic value of our scoring system, Kaplan–Meier analysis was applied in the training cohort. The 5-year survival curves of type 1 and type 2 showed better survival than those of type 3 and type 4 (Fig. 4a). To validate the prediction accuracy of our scoring system, the time-dependent AUC and ROC curves were employed for further evaluation (Fig. 4b, c). The time-dependent AUC was higher than 0.6, suggesting that the prediction model may be an independent factor in predicting the prognosis of TNBC. We found that the AUC was 0.706 in the 1st year, 0.607 in the 3rd year, and 0.678 in the 5th year, indicating the moderate ability of our system in prognosis prediction. In the training cohort, Kaplan–Meier analysis of OS suggested that the low-risk group had a better prognosis than the high-risk group (*P* < 0.05) (Fig. 4d). To confirm that the scoring system shared a similar prognostic value in different populations, we applied it to the validation cohorts. The result of the METABRIC cohort was consistent with that of the training cohort, disclosing that our scoring system was able to discriminate patients with better or worse OS (Fig. 4e). Though the difference did not reach statistical significance, the low-risk group showed a better OS in the GSE58812 cohort.

To further investigate the rationality and universality of our scoring system, an IHC assay was performed in the SYSUCC cohort (*N* = 51) to facilitate its clinical application. Based on the evaluation of CD4^+^ T cells, CD8^+^ T cells, and M2 macrophages, patients were classified into the above-mentioned 4 phenotypes and a consistent result could be achieved in the Kaplan–Meier analysis of OS (Figure S2a, b).

### Classification associated with immune pathways and checkpoint molecules

**Fig. 5:**
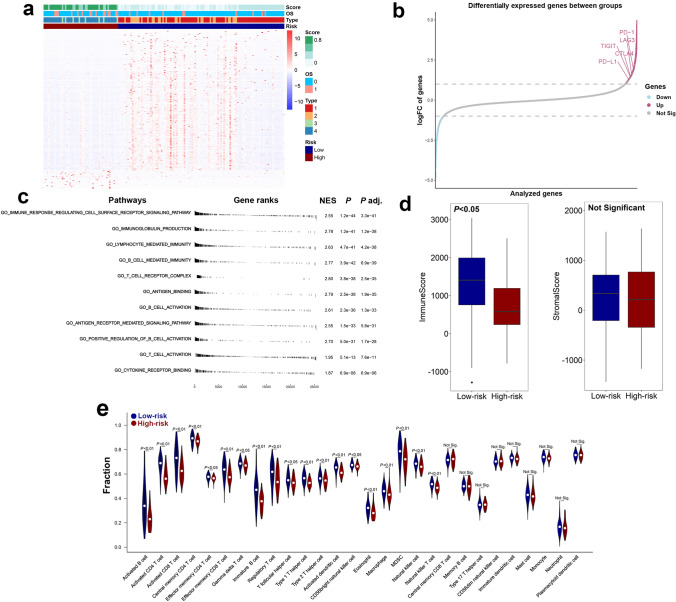
Immune characteristics of different risk groups. **a** Heatmap shows the correlation between risk score, prognosis, and the expressions of immune-related genes. A total of 404 differentially expressed genes were included in this analysis. The screening criteria of genes were: FDR < 0.05 and |logFC|> 1. A Chi-square test was adopted for data analysis. **b** Differentially expressed genes between the two groups. Compared with the high-risk group, several key immune checkpoint molecules that were upregulated in the low-risk group were pointed out. **c** GSEA pathway enrichment analyses of differentially expressed genes between the two groups. **d** The immune score and stromal score of the 2 groups were evaluated by the ESTIMATE algorithm. Blue and red represent the low-risk and high-risk groups, respectively. **e** Comparison of 28 infiltrating immune cells between the two groups. The CIBERSORT algorithm was employed. Blue and red represent the low-risk and high-risk groups, respectively

Further results showed the score of each patient and its correlation with the expressions of immune-related genes and clinical prognosis. A total of 404 differentially expressed genes were included in this analysis. Patients with lower scores expressed higher levels of immune-related genes, which indicated that these patients have a more active immune microenvironment. For overall survival, patients with higher scores seemed to have a higher rate of death, which indicates that the newly defined scoring system may be an independent and promising prognostic predictor for TNBC (Fig. 5a). Subsequent analyses proved that several key immune checkpoint molecules, PD-L1, PD-1, and CTLA-4, were significantly upregulated in TNBC patients with lower scores, demonstrating that the low-risk group might be more sensitive to immunotherapy. The expression of some immune-related molecules, such as LAG3 and TIGIT, was significantly higher in the low-risk group, indicating their roles as potential therapeutic targets in TNBC treatment (Fig. 5b). We then applied GSEA to examine the relevant signaling pathways involved in patients with low scores. Our results revealed that several key immune-related pathways were significantly enriched in the low-risk group, including immune response regulating cell surface receptor signaling, T cell and B cell activation, lymphocyte and B cell-mediated immunity, immunoglobulin production, cytokine receptor binding, T cell receptor complex, and antigen receptor-mediated signaling (Fig. 5c, S1). Moreover, the ESTIMATE method was employed to explore the overall TME status. A high immune score, but not the stromal score, was observed in the low-risk group (Fig. 5d). To further investigate the cooperative contribution of infiltrating immune cells between the two groups, the CIBERSORT algorithm was used to estimate the proportion of 28 immune cells in the TCGA cohort (Fig. 5e). Patients in the low-risk group presented a higher percentage of antitumoral immune cells, including activated B cells (*P* < 0.01), activated CD4^+^ T cells (*P* < 0.01), activated CD8^+^ T cells (*P* < 0.01), NK cells (*P* < 0.01), and activated dendritic cells (*P* < 0.01). Taken together, these results showed that there was a more active immune microenvironment in the low-risk group compared with the high-risk one.

## Discussion

TNBC is a group of heterogeneous tumors, including several distinct molecular subtypes [23]. So far, a variety of genomic classifications and subtypes have been proposed. Due to the remarkable progress in immunotherapy, one of the most widely accepted subtypes is immune-enriched [2]. How to identify this subgroup of patients in clinical practice remains unresolved. The current study comprehensively analyzed 64 cell types of the TME and found that infiltrating with a high density of CD4^+^ memory T cells, CD8^+^ T cells, and Th2 cells were associated with superior survival while infiltrating with more M2 macrophages, endothelial cells, and mv endothelial cells contribute to worse survival. We aimed to establish a scoring system that can more comprehensively reflect the biological characteristics of the tumor microenvironment. However, due to the poor prognostic prediction ability of Th2 cells and the difficulty in distinguishing endothelial cell subtypes, an easy-to-use prognostic model with 3 cell types was built up in this study. Based on the scores of M2 macrophages, CD8^+^ T cells, and CD4^+^ memory T cells, patients with TNBC were divided into 4 phenotypes and 2 groups.

As it is well known, CD4^+^ Th1 cells and CD8^+^ T cells are the two main components of TILs [32]. CD4^+^ Th1 cells secrete IL-2 and IFN to activate and promote the proliferation of CD8^+^ T cells, which subsequently release cytotoxic cytokines and kill cancer cells directly [18]. Accumulating data prove that CD4^+^ Th1 cells and CD8^+^ T cells are associated with better survival both in the early-stage and metastatic TNBC, which may serve as a promising marker for identifying patients who are more likely to benefit from ICIs [7, 13, 25, 27]. As a typically pro-tumorigenic cell, M2 macrophage plays an immunosuppressive role in the TME by inhibiting the activation of M1 macrophage and secreting IL-10 and TGF-β [10]. Current evidence supports the negative prognostic role of M2 macrophages in breast cancer [4]. Tumor endothelial cells may provide activation signals or secrete biglycan to stimulate cancer cell metastasis. Recent studies have emphasized that “angiocrine factors” released by tumor endothelial cells can enhance the invasiveness of tumor cells [26]. Herein, excluding endothelial cells and mv endothelial cells from our model compromised its accuracy and reliability, which was one of our limitations.

During the Kaplan–Meier analysis, a distinct difference in survival was observed among different subtypes, which was further confirmed in the SYSUCC cohort. The low-risk group exhibited a better prognosis than the high-risk one in the training cohort, but there was a slight difference in the GSE58812 cohort (*P* = 0.088). Part of the explanation was that the sample size of this cohort was relatively small (*N* = 107), especially when it was classified into 2 groups. And the moderate predictive ability of our 3 cell types model might be one reason that accounted for this finding. Through an in-depth exploration of the differentially expressed genes of the 2 groups, we found that the immune microenvironment of the low-risk group was more active, with immune-related pathways enriched and immune checkpoint molecules upregulated. At the same time, a higher percentage of anti-tumor immune cells were infiltrated in the TME of the low-risk group, implying there was both a consuming anti-cancer immune response and an increased anti-immune response in this microenvironment. The active anti-cancer immune response might be restored by that application of immune checkpoint blockade, such as anti-PD-1/PD-L1 treatment.

As previous studies reported, approximately 20% of TNBCs were classified as immunomodulatory (IM), in which immune cell markers and signalings, such as NFkB, TNF, JAK, and cytokine signaling, were highly enriched. Through histological assessment, RNA detection, and gene expression analysis, it was proven that the IM subtype was one kind of tumor with substantial infiltrating lymphocytes, which owned a better prognosis compared with other subtypes of TNBC [24]. Besides, more immune checkpoint molecular gene expressions, such as PD-1, PD-L1, and CTLA-4, were found in the TME of IM subtype, which was quite similar to the low-risk group of our study [5].

Compared with the heterogeneity in PD-L1 expression, this immune infiltrated cell-based scoring system could better reflect the immune status of the TME, thus more accurately identifying the population with stronger immune activity against tumor cells [22]. Besides, there was no apparent correlation between the level of immune cell infiltration and that of PD-L1 expression in the TME. Herein, combining two or more methods to capture characteristics of the TME may be more effective as a comprehensively predictive indicator for immunotherapy [16]. In a meta-analysis, biomarkers such as PD-L1 expression combined with tumor mutational burden (TMB) had been demonstrated an advantageous performance than PD-L1 expression or TMB alone [17]. Whether the combination of TMB and our classifier has superior performance still needs to be further confirmed in future studies.

The study has the following limitations. First of all, this study is a retrospective analysis of public data sets that lack clinical information to improve their accuracy and cannot eliminate the heterogeneity between different populations. Therefore, prospective studies are needed to verify the predictive value of our scoring system, especially in terms of immunotherapy response prediction. Furthermore, the selected cells were not analyzed at all tumor sites due to the unavailability of pathological slides from the common data set, and the tissue microarray only reflected the average cellular composition. Additionally, only 3 types of prognostic-related cells were included in the model. There may be other more comprehensive panels that we missed. Subsequent inclusion of some stromal cells may more fully reflect the features of TME and minimize the potential of bias.

## Conclusion

In conclusion, a risk scoring system based on 3 cell types of the TME was developed and validated, which could cluster patients with TNBC into 4 phenotypes and 2 groups. And the low-risk group has a superior prognosis and more active immune activity than the high-risk one. Though this novel scoring system might predict the survival, immune activity, and potential therapeutic response of patients, we recommend further validating its efficacy in prospective studies.

## Supplementary Information

Below is the link to the electronic supplementary material.

Supplementary file2 Figure S2. (a) Representative microphotographs showing of CD8+ T cells, CD4+ T cells, and M2 macrophages using IHC assay. (b) The Kaplan-Meier analyses of OS among the 4 phenotypes in the SYSUCC cohort. (TIF 15880 kb)

Supplementary file3 Table S1. Antibodies were used in this study. (DOCX 13 kb)

Supplementary file1 Figure S1. GSEA analysis revealed 11 pathways were associated with differentially expressed genes between the low-risk and high-risk groups (PDF 410 kb)

## Data Availability

The datasets generated for this study are available on request to the corresponding author.
