# Supplementary figures and images for: A risk scoring system based on tumor microenvironment cells to predict prognosis and immune activity in triple-negative breast cancer
Source: Breast Cancer. 2022 Jan 21;29(3):468–77. doi: 10.1007/s12282-021-01326-w (PMC9021102; doi:10.1007/s12282-021-01326-w)

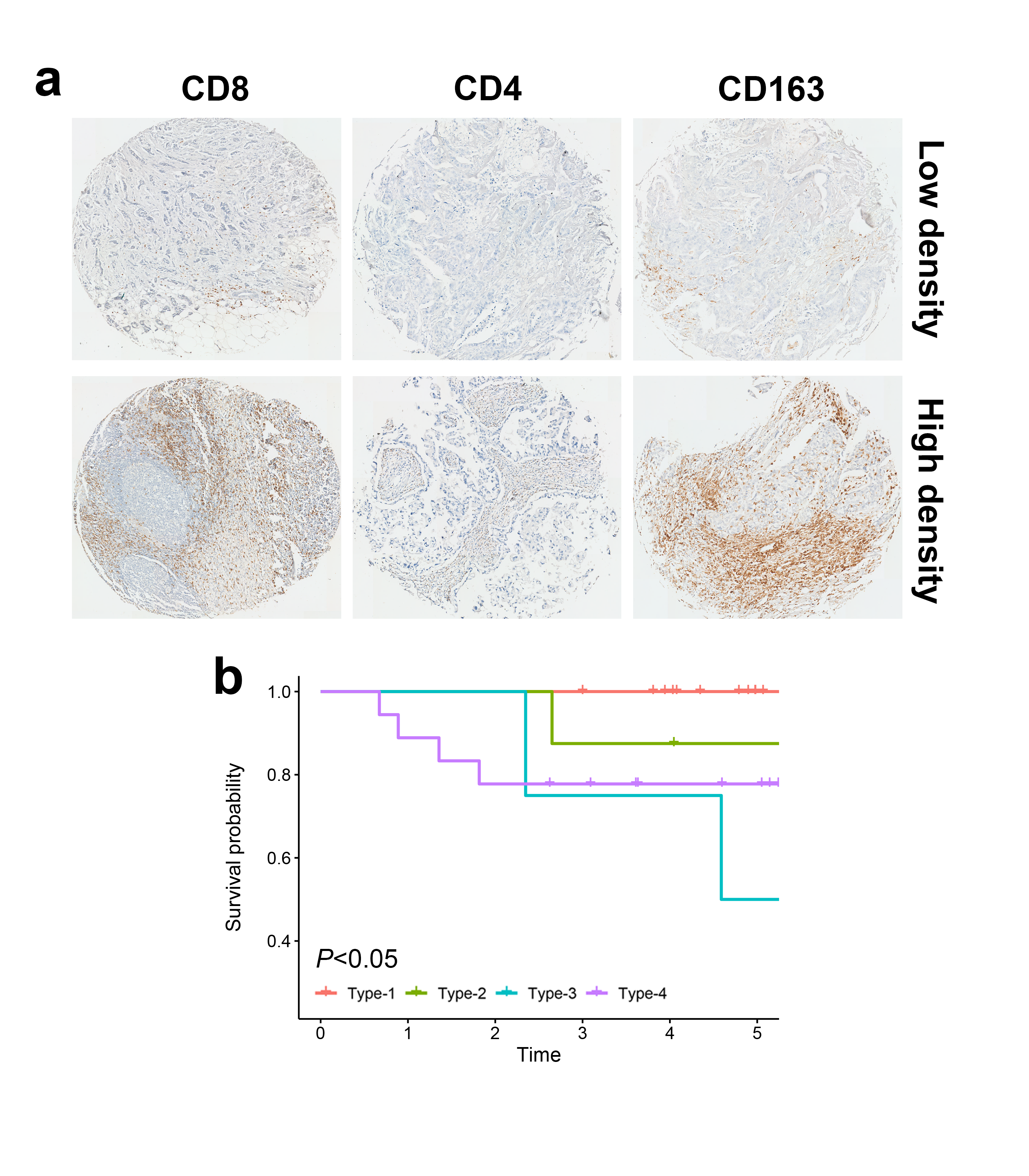

Supplement: Supplementary file 1 — Supplementary file2 Figure S2. (a) Representative microphotographs showing of CD8+ T cells, CD4+ T cells, and M2 macrophages using IHC assay. (b) The Kaplan-Meier analyses of OS among the 4 phenotypes in the SYSUCC cohort. (TIF 15880 kb) [file 12282_2021_1326_MOESM1_ESM.tif]
