# Supplementary material for: A risk scoring system based on tumor microenvironment cells to predict prognosis and immune activity in triple-negative breast cancer
Source: Breast Cancer. 2022 Jan 21;29(3):468–77. doi: 10.1007/s12282-021-01326-w (PMC9021102; doi:10.1007/s12282-021-01326-w)
Supplement: Supplementary file 2 — Supplementary file3 Table S1. Antibodies were used in this study. (DOCX 13 kb) [file 12282_2021_1326_MOESM2_ESM.docx]

| Targets | Markers | Source | Dilution |
| --- | --- | --- | --- |
| CD4+T cells | CD4 | ZSGB-BIO | Ready to use |
| CD8+T cells | CD8 | ZSGB-BIO | Ready to use |
| Macrophage M2 | CD163 | ZSGB-BIO | Ready to use |
