# Supplementary material for: A risk scoring system based on tumor microenvironment cells to predict prognosis and immune activity in triple-negative breast cancer
Source: Breast Cancer. 2022 Jan 21;29(3):468–77. doi: 10.1007/s12282-021-01326-w (PMC9021102; doi:10.1007/s12282-021-01326-w)
Supplement: Supplementary file 3 — Supplementary file1 Figure S1. GSEA analysis revealed 11 pathways were associated with differentially expressed genes between the low-risk and high-risk groups (PDF 410 kb) [file 12282_2021_1326_MOESM3_ESM.pdf]

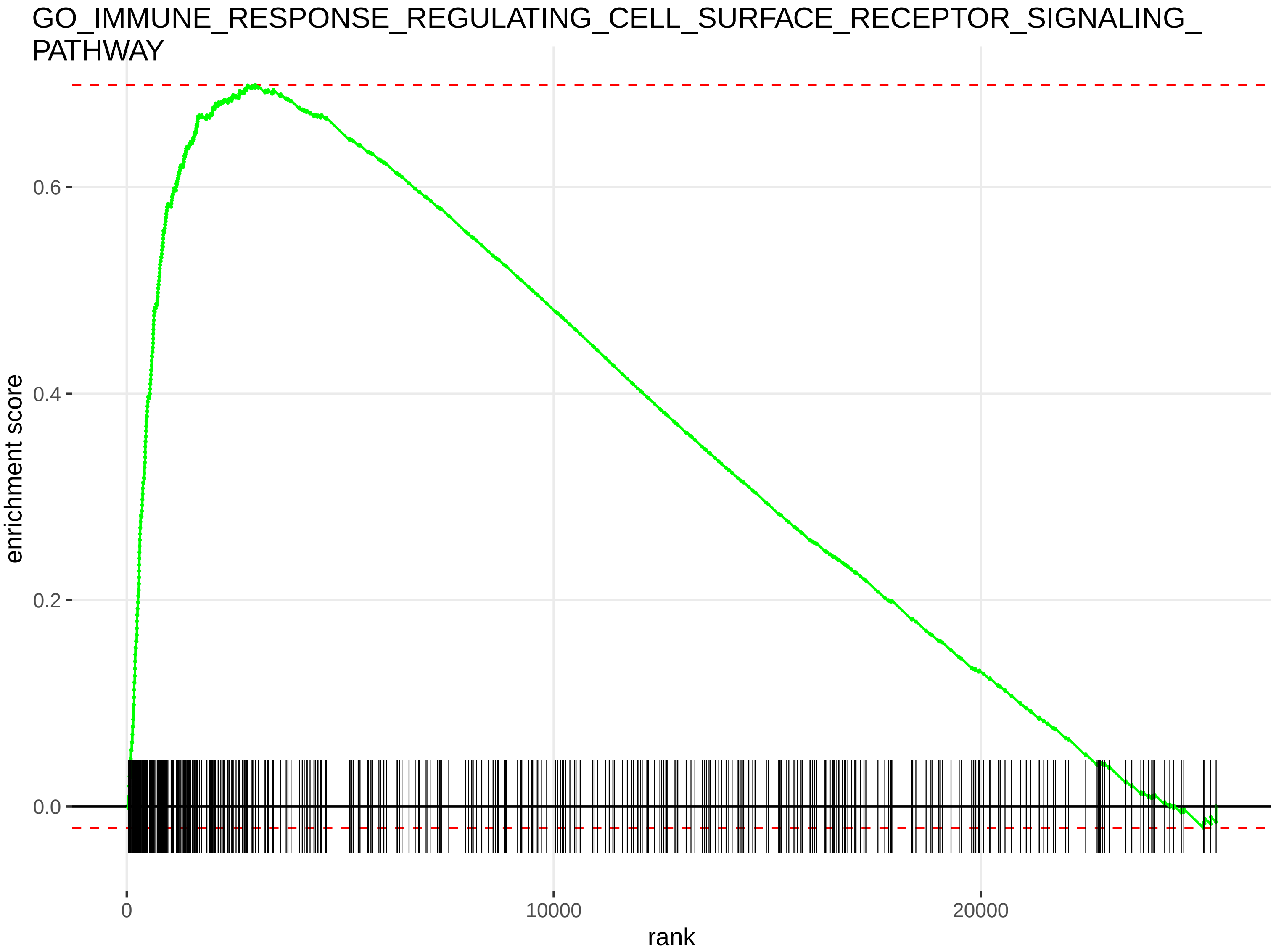
GO_IMMUNE_RESPONSE_REGULATING_CELL_SURFACE_RECEPTOR_SIGNALING_
PATHWAY
enrichment score
0.6
0.4
0.2
0.0
0
10000
20000
rank

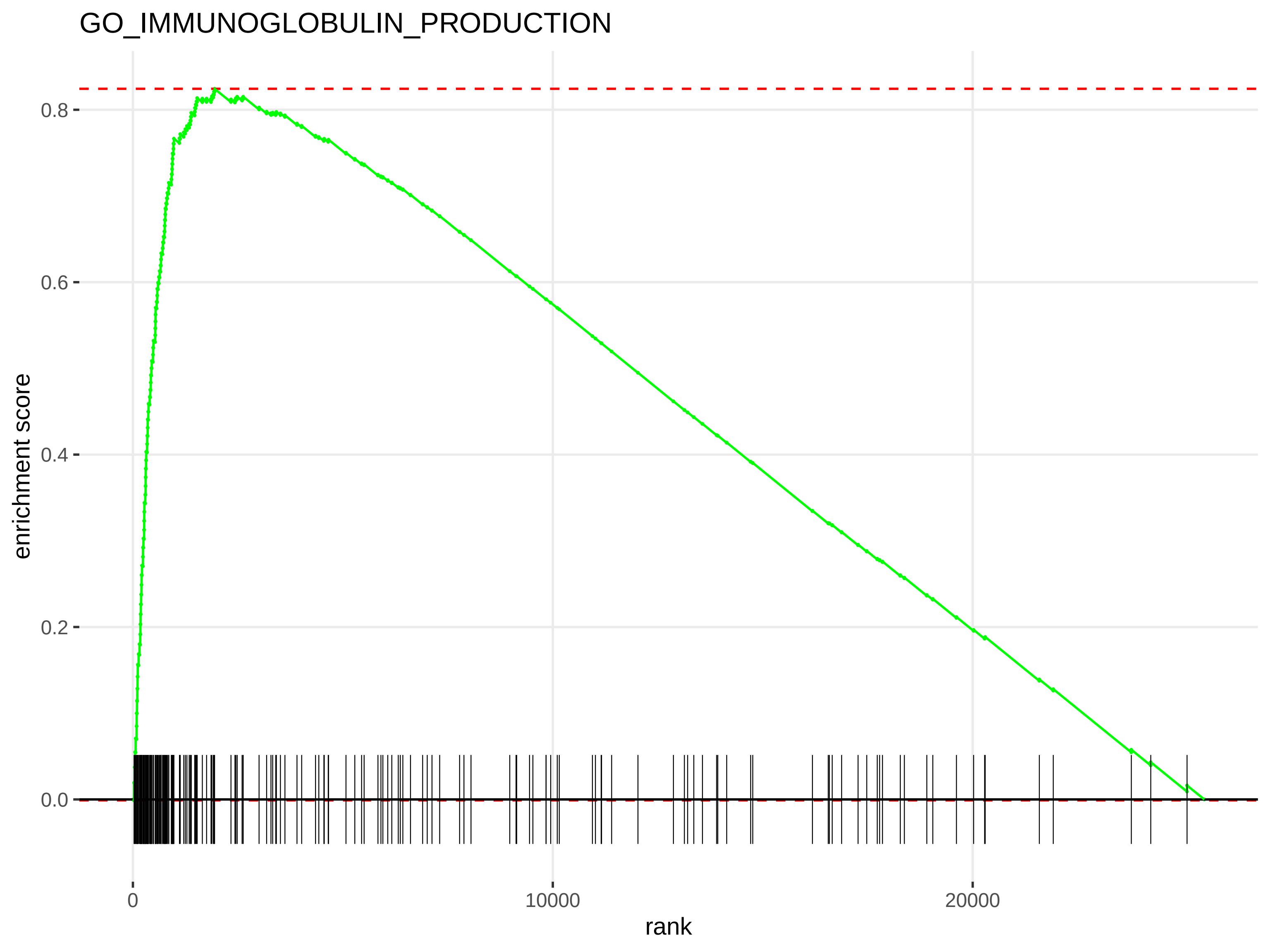

GO_IMMUNOGLOBULIN_PRODUCTION
0.8
0.6
0.4
0.2
0.0
enrichment score
0
10000
20000
rank

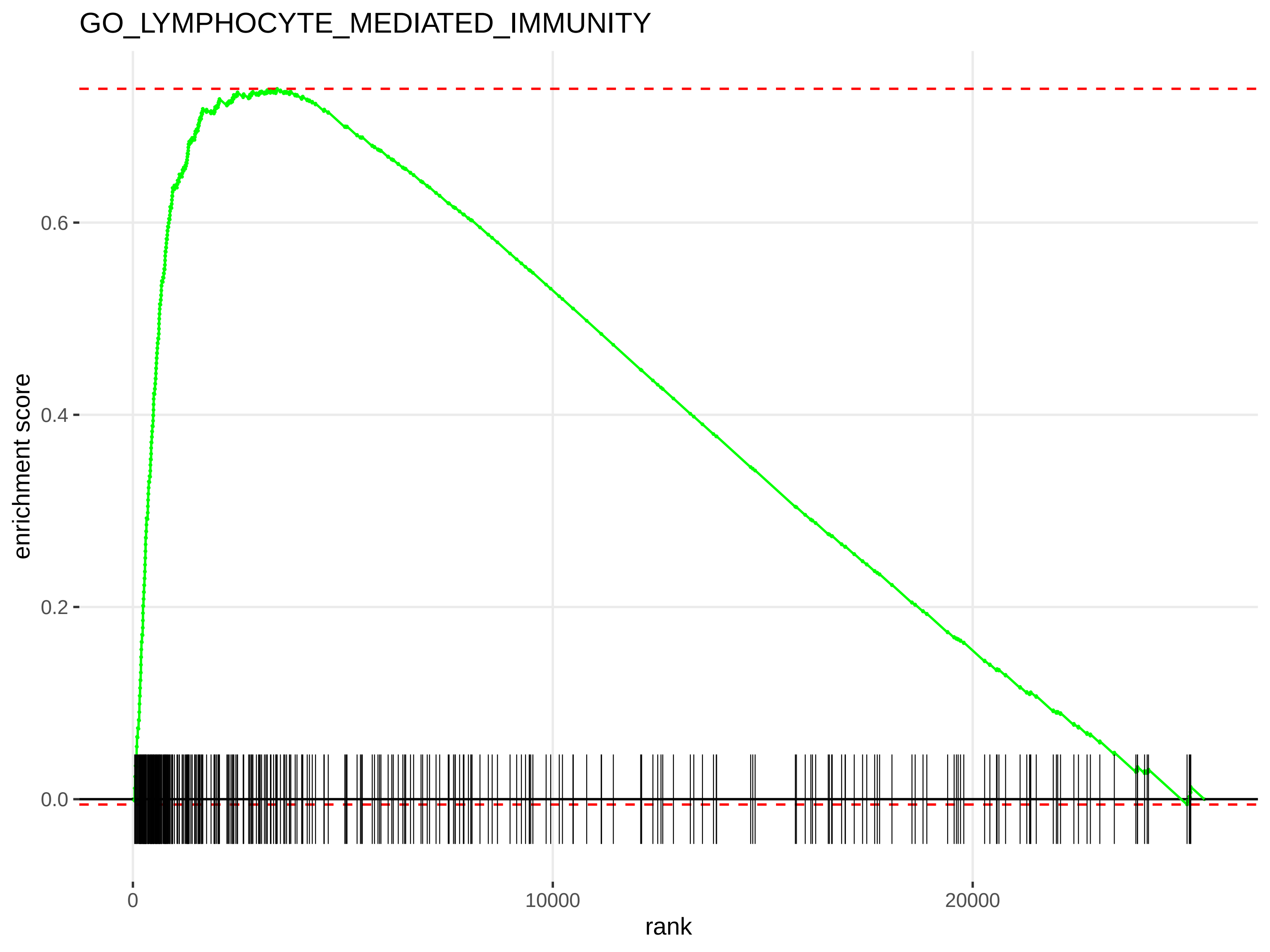
GO_LYMPHOCYTE_MEDIATED_IMMUNITY
enrichment score
0.6
0.4
0.2
0.0
0
10000
20000
rank

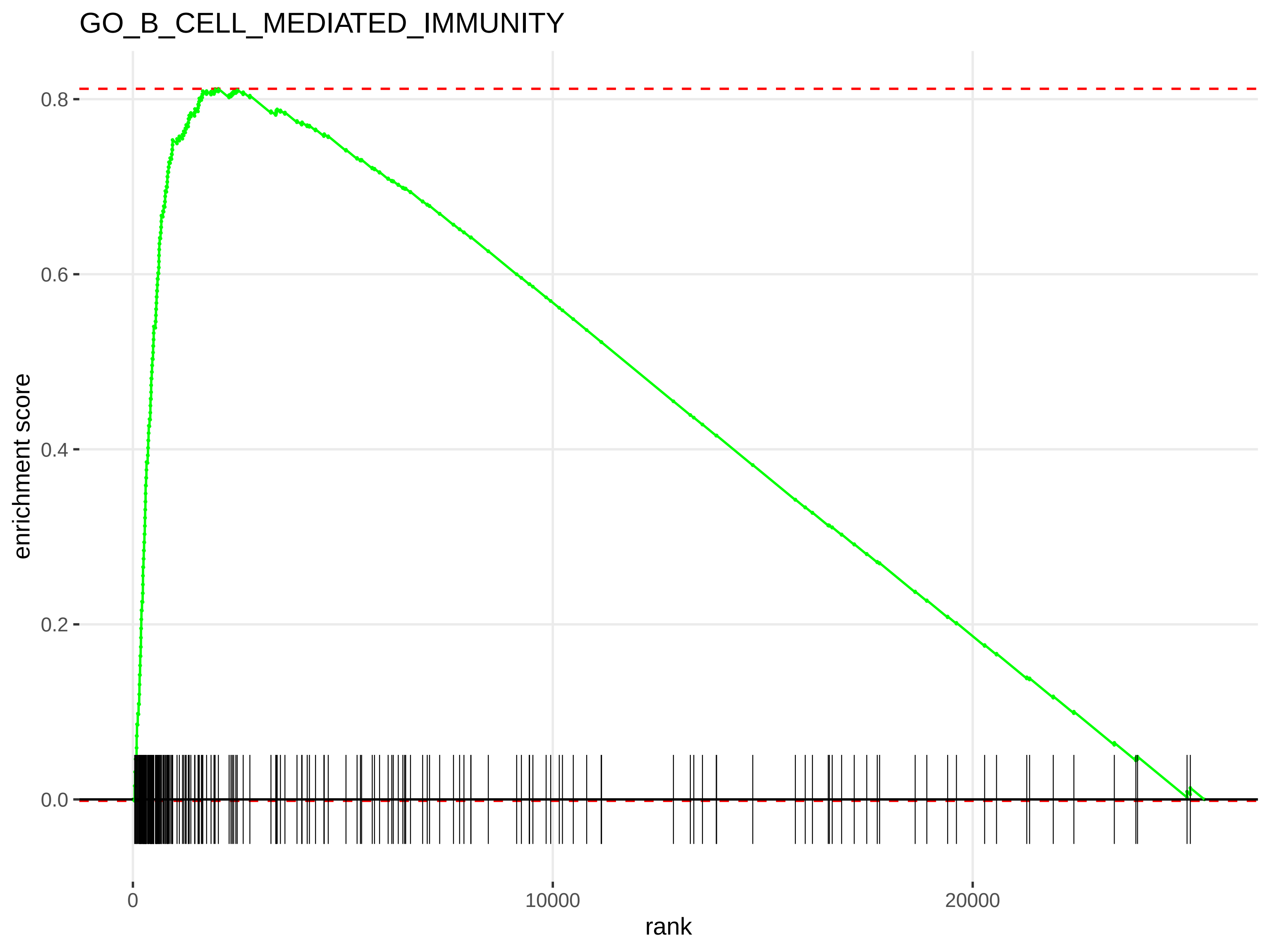
GO_B_CELL_MEDIATED_IMMUNITY
enrichment score
0.8
0.6
0.4
0.2
0.0
0
10000
20000
rank

GO_T_CELL_RECEPTOR_COMPLEX

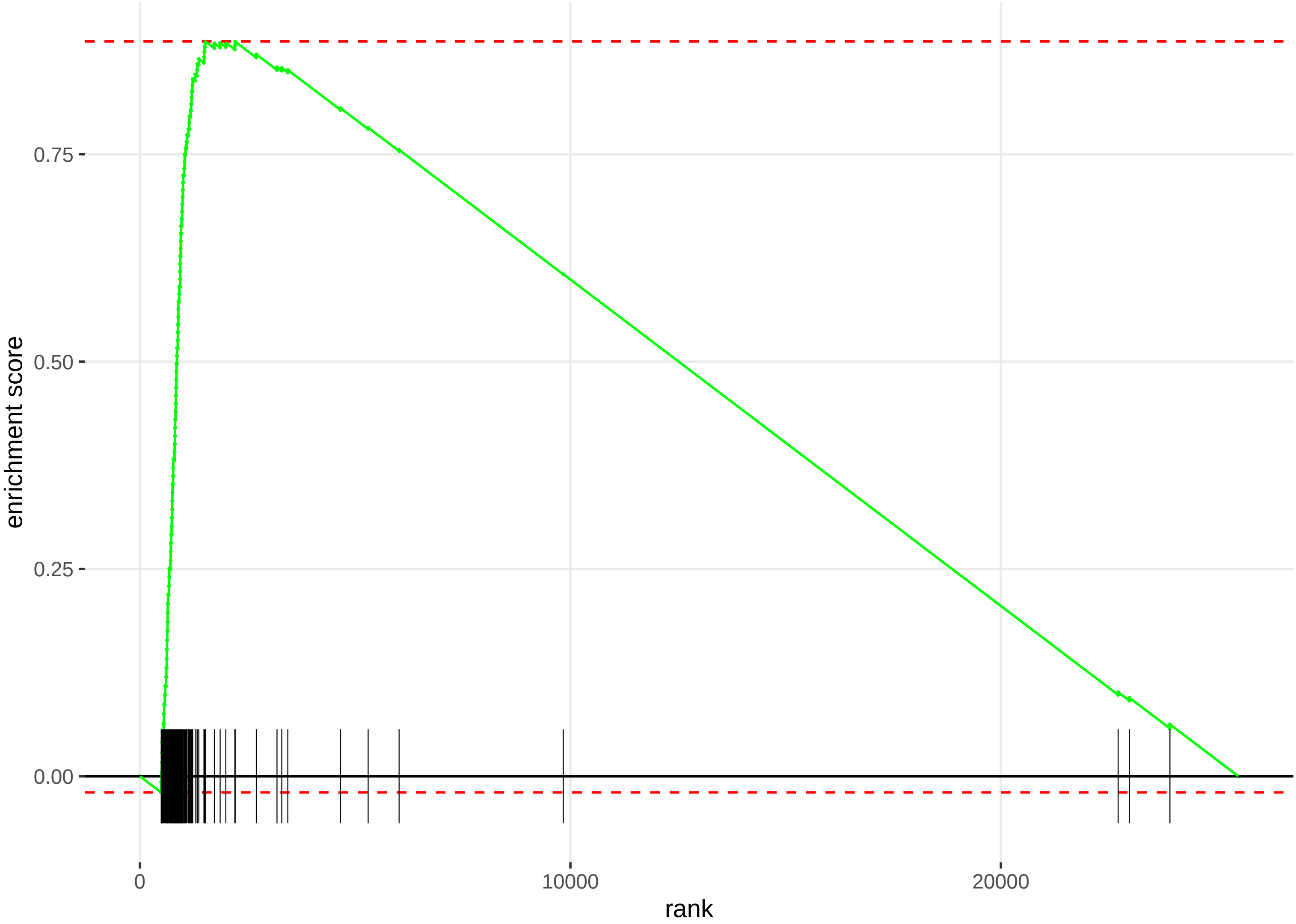

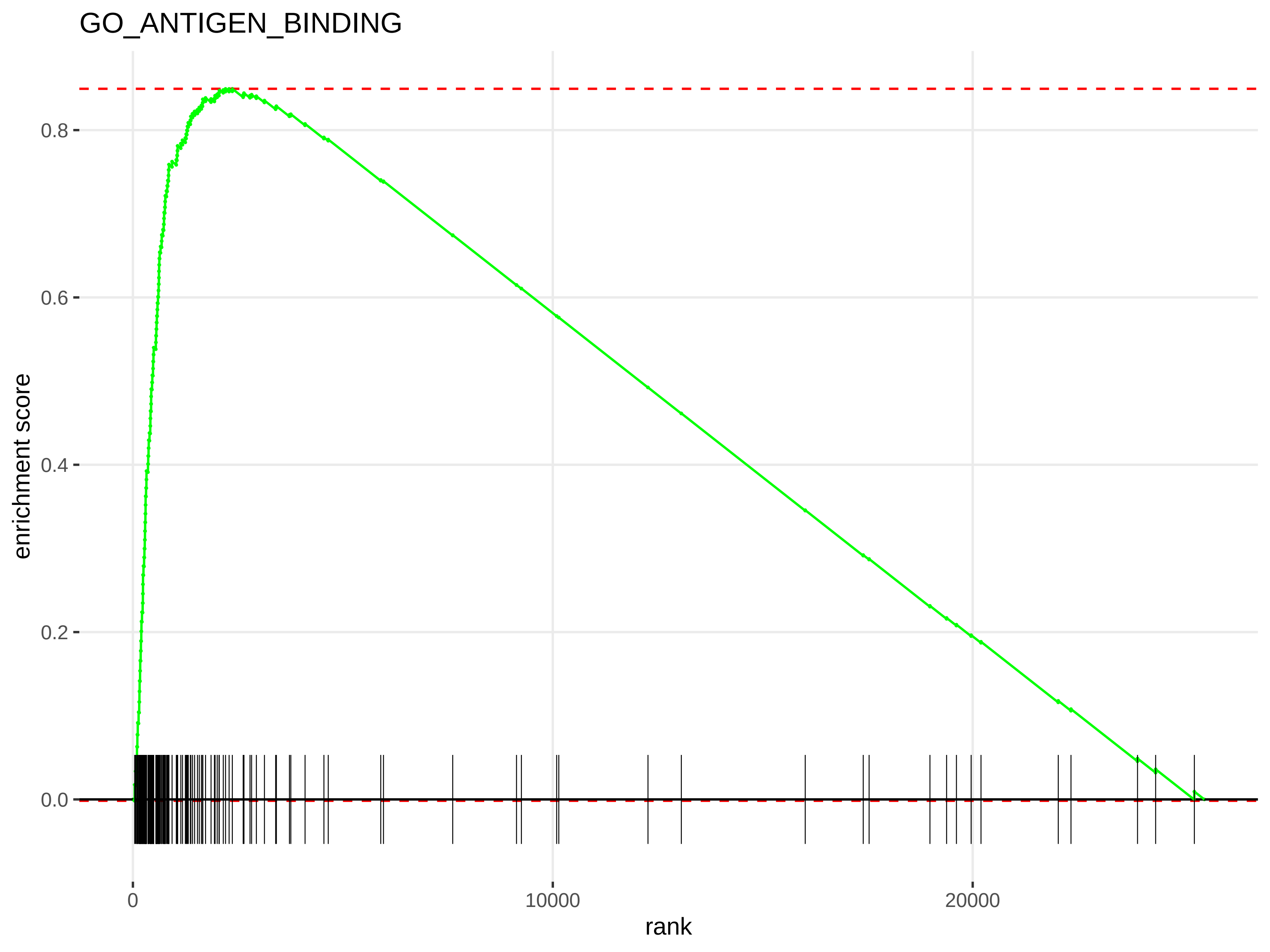
GO_ANTIGEN_BINDING
enrichment score
0.8
0.6
0.4
0.2
0.0
0
10000
20000
rank

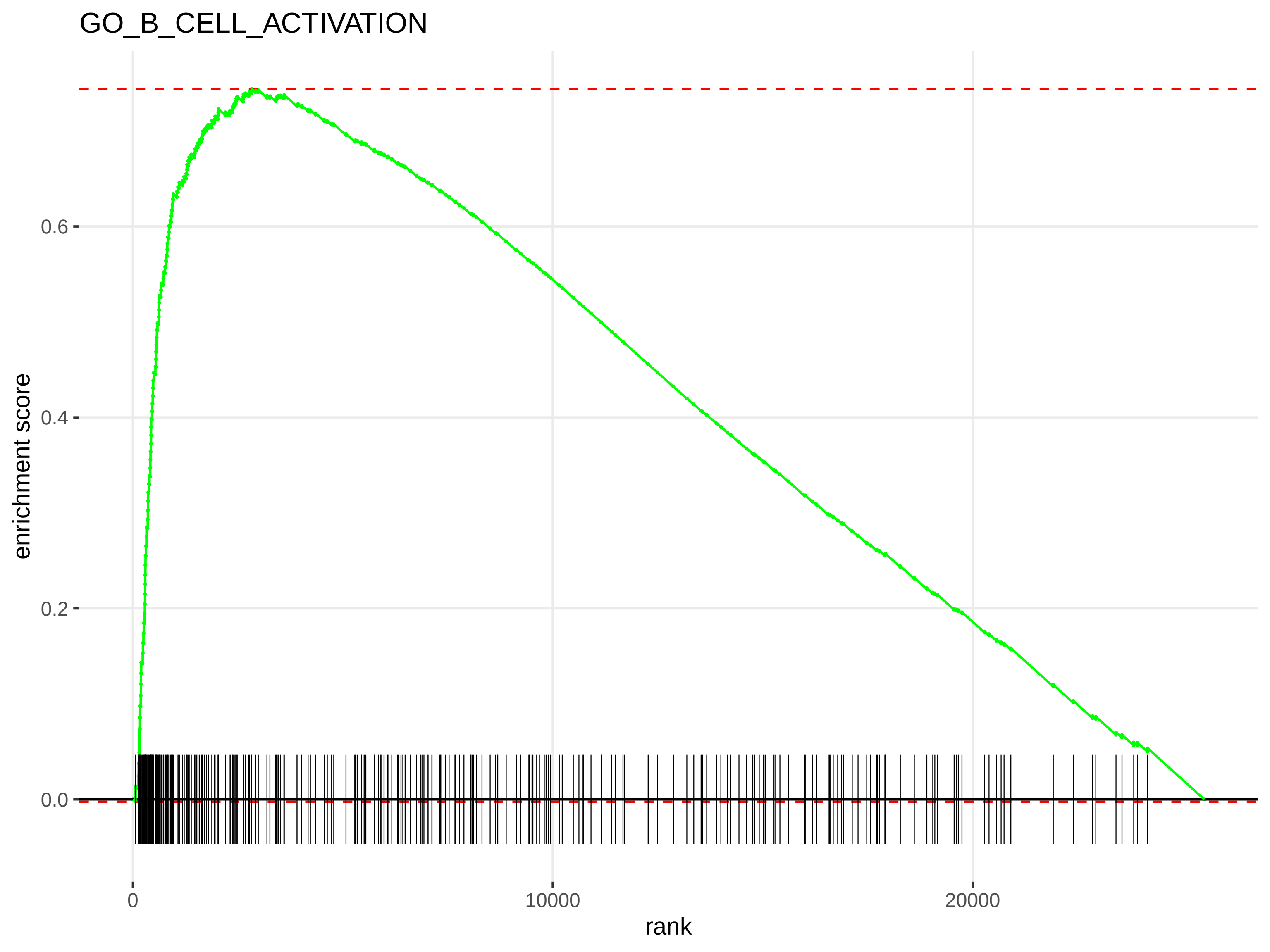

GO_B_CELL_ACTIVATION
enrichment score
0.0
0.2
0.4
0.6
0
10000
20000
rank

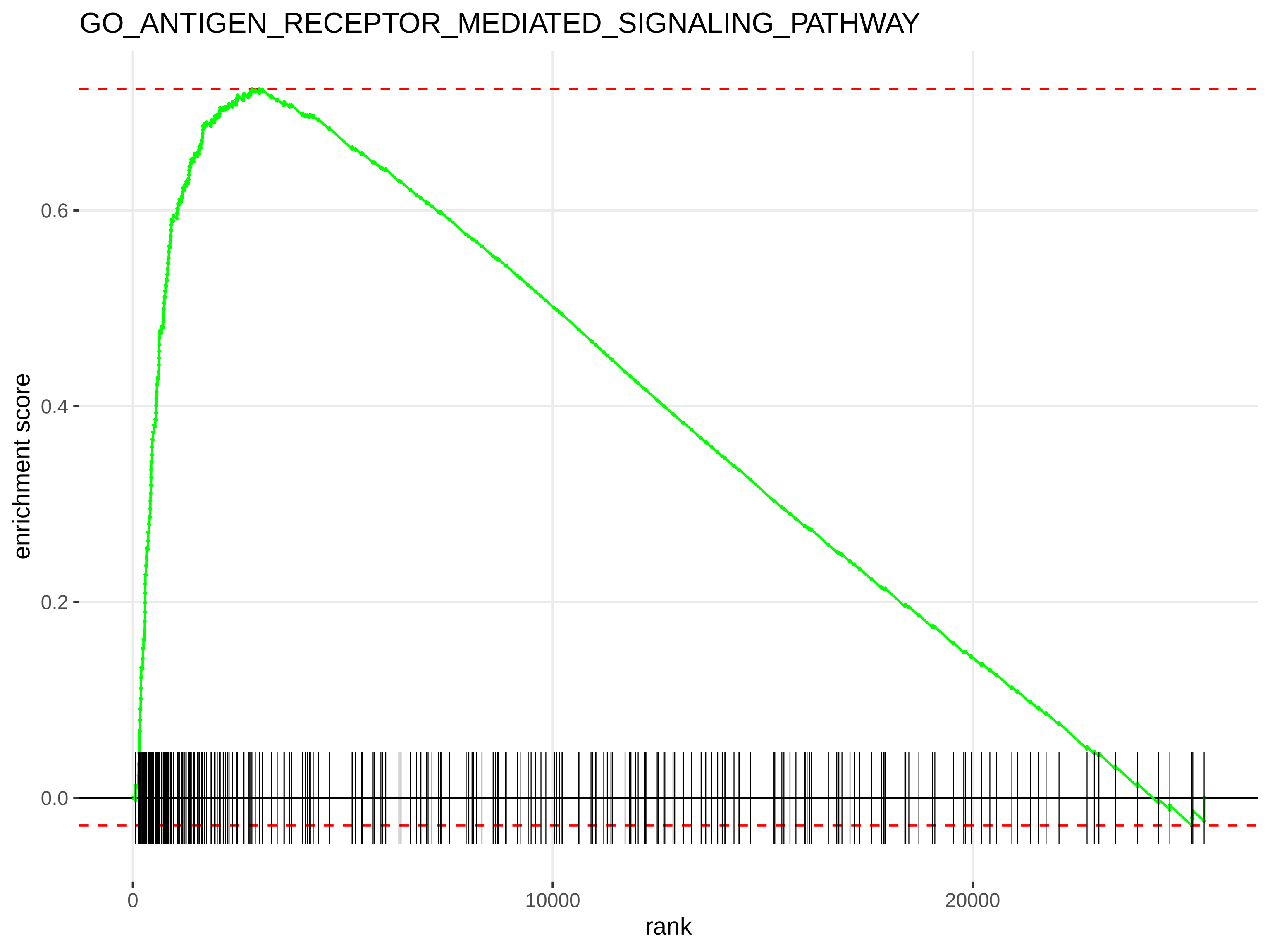
GO_ANTIGEN_RECEPTOR_MEDIATED_SIGNALING_PATHWAY
enrichment score
0.6
0.4
0.2
0.0
0
10000
20000
rank

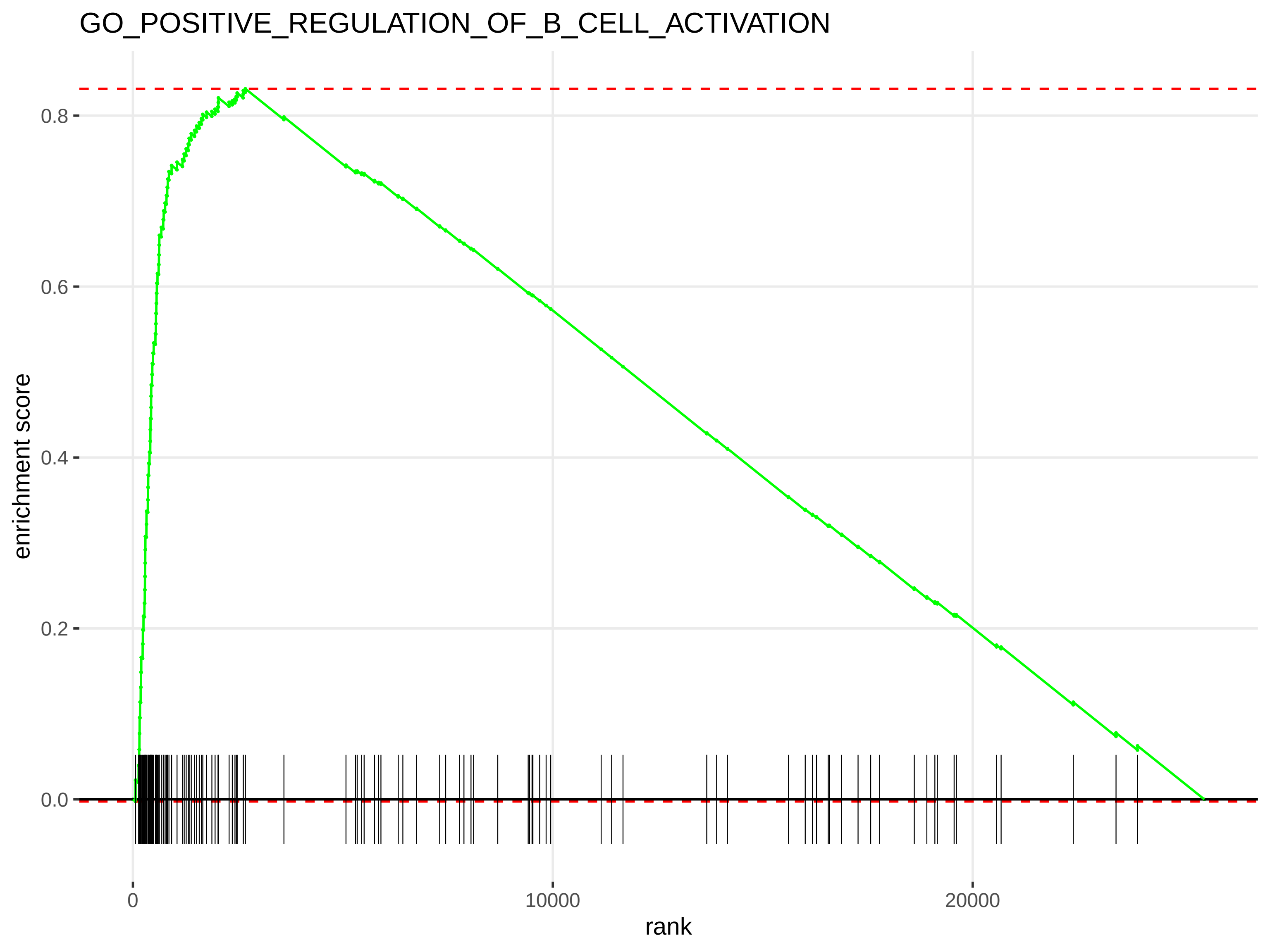
GO_POSITIVE_REGULATION_OF_B_CELL_ACTIVATION
enrichment score
0.8
0.6
0.4
0.2
0.0
0
10000
20000
rank

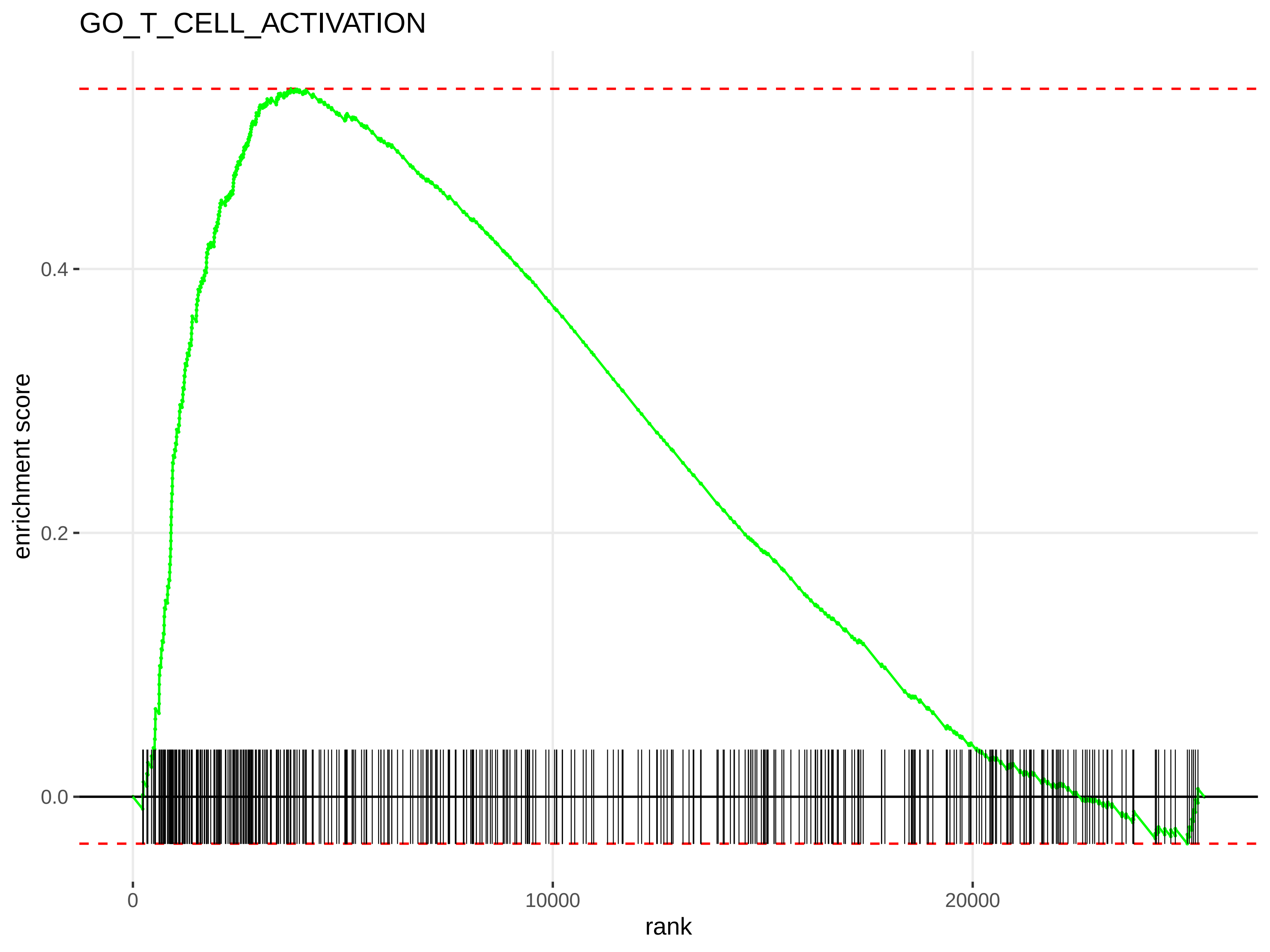

GO_T_CELL_ACTIVATION
enrichment score
0.4
0.2
0.0
0
10000
20000
rank

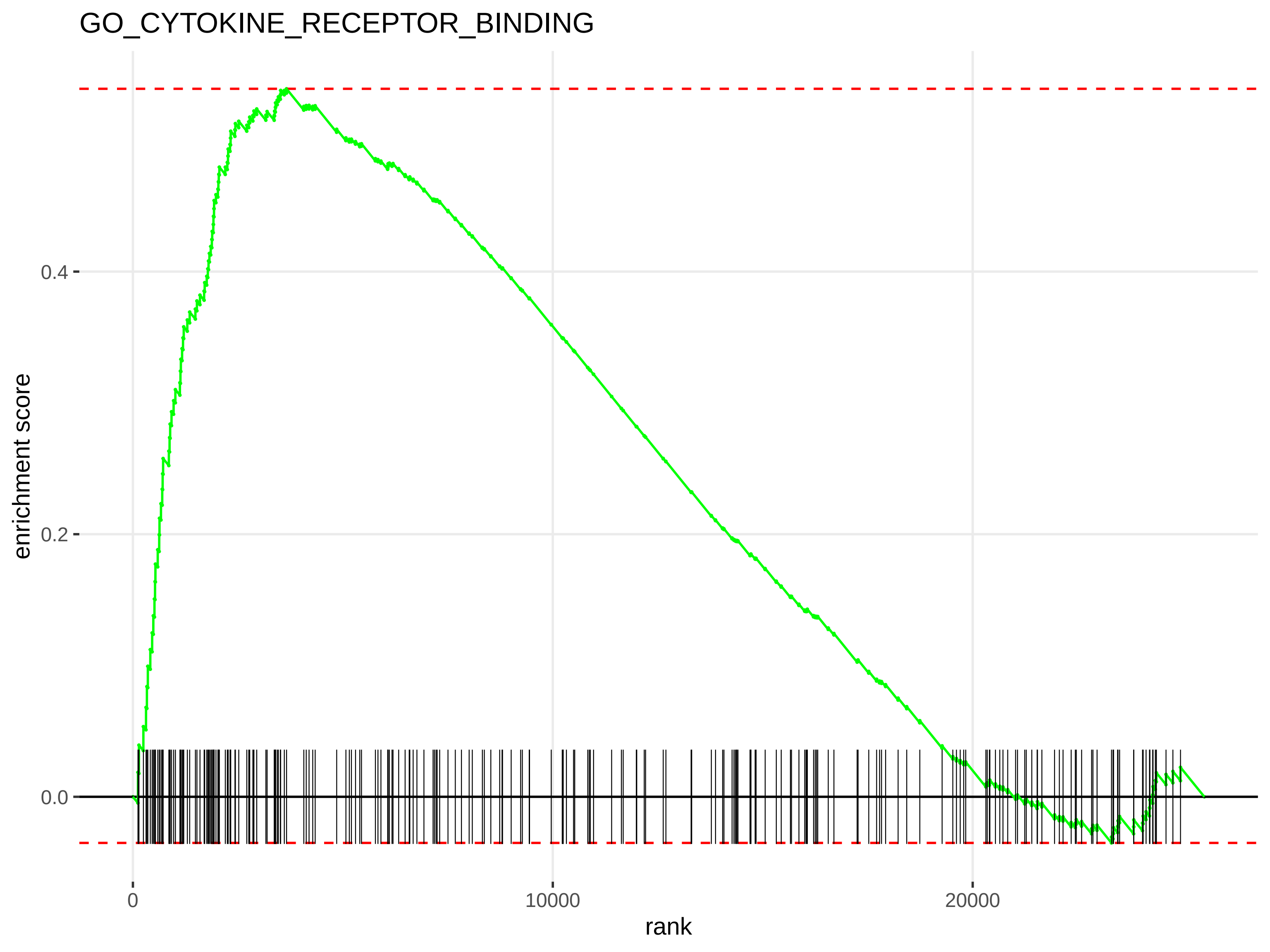
GO_CYTOKINE_RECEPTOR_BINDING
enrichment score
0.4
0.2
0.0
0
10000
20000
rank
